# ANIA: ANnotation and Integrated Analysis of the 14-3-3 interactome

**DOI:** 10.1093/database/bat085

**Published:** 2014-02-05

**Authors:** Michele Tinti, Fábio Madeira, Gavuthami Murugesan, Gerta Hoxhaj, Rachel Toth, Carol MacKintosh

**Affiliations:** ^1^MRC Protein Phosphorylation Unit, College of Life Sciences, University of Dundee, Dundee DD1 5EH, Scotland, UK and ^2^Division of Cell and Developmental Biology, College of Life Sciences, University of Dundee, Dundee DD1 5EH, Scotland, UK

## Abstract

The dimeric 14-3-3 proteins dock onto pairs of phosphorylated Ser and Thr residues on hundreds of proteins, and thereby regulate many events in mammalian cells. To facilitate global analyses of these interactions, we developed a web resource named ANIA: ANnotation and Integrated Analysis of the 14-3-3 interactome, which integrates multiple data sets on 14-3-3-binding phosphoproteins. ANIA also pinpoints candidate 14-3-3-binding phosphosites using predictor algorithms, assisted by our recent discovery that the human 14-3-3-interactome is highly enriched in 2R-ohnologues. 2R-ohnologues are proteins in families of two to four, generated by two rounds of whole genome duplication at the origin of the vertebrate animals. ANIA identifies candidate ‘lynchpins’, which are 14-3-3-binding phosphosites that are conserved across members of a given 2R-ohnologue protein family. Other features of ANIA include a link to the catalogue of somatic mutations in cancer database to find cancer polymorphisms that map to 14-3-3-binding phosphosites, which would be expected to interfere with 14-3-3 interactions. We used ANIA to map known and candidate 14-3-3-binding enzymes within the 2R-ohnologue complement of the human kinome. Our projections indicate that 14-3-3s dock onto many more human kinases than has been realized. Guided by ANIA, PAK4, 6 and 7 (p21-activated kinases 4, 6 and 7) were experimentally validated as a 2R-ohnologue family of 14-3-3-binding phosphoproteins. PAK4 binding to 14-3-3 is stimulated by phorbol ester, and involves the ‘lynchpin’ site phosphoSer99 and a major contribution from Ser181. In contrast, PAK6 and PAK7 display strong phorbol ester-independent binding to 14-3-3, with Ser113 critical for the interaction with PAK6. These data point to differential 14-3-3 regulation of PAKs in control of cell morphology.

**Database URL**: https://ania-1433.lifesci.dundee.ac.uk/prediction/webserver/index.py

## Introduction

The eukaryotic 14-3-3 family of phosphoprotein-binding proteins exert pleiotropic regulatory effects by docking onto a constellation of intracellular targets. Collating the cumulative data from low- (LT) and high-throughput (HT) studies of 14-3-3-binding phosphoproteins has given valuable insights into 14-3-3 specificities, and how 14-3-3s regulate cellular responses to growth factors and other stimuli (1–3). Identifying patterns in the global data also led to exciting discoveries about how the 14-3-3-interactome was shaped by major evolutionary changes in the vertebrate animals, and highlighted that 14-3-3s interact with many proteins that are deregulated in cancer, and metabolic and neurological disorders (1,4). To facilitate further discoveries, here we developed ANIA, a web resource for ANnotation and Integrated Analysis of the 14-3-3 interactome.

The 14-3-3s are dimers in which two curved L-shaped monomers form a boat shape around an ∼35 Å wide inner groove that has a phosphoSer/Thr-binding pocket at either side. 14-3-3 dimers therefore dock onto pairs of phosphosites, and these are usually in tandem on the same target. The paired sites sometimes lie close to or either side of a domain or motif in the target, which is occluded by the bound 14-3-3. In other cases, the two phosphosites lie within a disordered region where the 14-3-3 can impose a conformational change on the target (5–8).

The binding specificity of 14-3-3s is determined by overall steric fit and by the linear sequence around the phosphorylated binding sites. Screens of synthetic phosphopeptides showed that mode I RSX(pS)XP and mode II RX(F/Y)X(pS)XP sequences display optimal binding to 14-3-3s (8,9). These data were used as the basis of the 14-3-3-phosphosite prediction algorithm within Scansite (9). When >200 experimentally determined 14-3-3-binding sites [dubbed ‘gold standards’ (GDs)] were analysed, variants on the mode I RSX(pS/pT)XP motifs were found to dominate, although the +2 proline residue occurs in less than half (5) including the mode III sites where the 14-3-3-binding phosphosite is the penultimate C-terminal residue. Around half of the sites also had a phosphorylatable serine at residue −2 relative to the phosphosite that binds to the 14-3-3 (1,10). While the protein kinases responsible for creating 14-3-3-binding phosphosites are known for only a limited number of cases, 14-3-3-binding sequences generally conform to the types of motifs generated by different basophilic protein kinase A/protein kinase G/protein kinase C family (AGC) and Ca^2+^/calmodulin-dependent protein kinase (CaMK) protein kinases (5,11). Consistent with these observations, 14-3-3-phosphoprotein interactions mediate cellular responses to insulin, growth factors and other stimuli that activate these kinases (12–14).

Remarkably, the GD human 14-3-3-binding phosphoproteins were recently discovered to be highly enriched in 2R-ohnologues. 2R-ohnologues are members of families of two to four proteins that were generated by two rounds of whole genome duplication (2R-WGD) at the origins of the vertebrate animals during the Cambrian period (15). While most of the quadruplicated DNA reverted to the singleton state, the retained 2R-WGD protein-coding gene families are highly enriched in signaling proteins in general (16), and 14-3-3-binding phosphoproteins in particular (1).

Human 2R-ohnologue families were identified whose members share one conserved ‘lynchpin’ 14-3-3-binding phosphosite. Lynchpins also align with a Ser/Thr residue in the single pro-orthologue proteins of the invertebrate chordates, namely *Branchiostoma* (Cephalochordata) and *Ciona* (tunicates), which are proxy for the pre-2R-WGD ancestor. In contrast, the second 14-3-3-binding sites may differ in different family members (1). These phosphorylation patterns inspired the hypothesis that 14-3-3 dimers may have helped shape the evolutionary divergence of their target 2R-ohnologue families. It is proposed that binding of 14-3-3s to conserved lynchpin phosphosites provided sufficient regulation that secondary sites in different family members were freer to evolve into sites that are phosphorylated by different kinases and exhibit different affinities for 14-3-3s. The resulting 2R-ohnologue families therefore operate as ‘signal multiplexing’ systems that integrate more regulatory inputs than the single ancestral protein, providing the increased signaling complexity needed for vertebrate life (1,16,17). To test the lynchpin and signal multiplexing hypotheses further and explore their biomedical implications, there is a pressing need to map the 14-3-3-interactome and 14-3-3-binding phosphosites systematically.

As well as the GD 14-3-3-binding phosphoproteins, a growing number of proteins have been isolated in HT 14-3-3-affinity capture experiments, and identified by mass spectrometric methods (2,12–14). These proteins likely include phosphoproteins that bind directly to 14-3-3s, proteins that bind indirectly to 14-3-3s as components of multi-subunit complexes and proteins that have been isolated non-specifically (though the HT experiments use strategies to enhance specificity). Given the striking enrichment of 2R-ohnologues among the GD 14-3-3-binding phosphoproteins (1), we surmise that 2R-ohnologues would make a useful priority list for validation of targets identified in HT experiments. Using 2R-ohnologue sequence alignments and bioinformatics methods to search for potential ‘lynchpins’ and second sites should also accelerate efforts to pinpoint regulatory 14-3-3-binding sites.

Here, we therefore generated ANIA, which integrates GD and HT data sets of 14-3-3-binding phosphoproteins, sorts the proteins into 2R-ohnologue families and uses a prediction algorithm based on the GD 14-3-3-binding phosphosites (unpublished work by Michele Tinti and the group of Geoff Barton) as well as sequence conservation analysis to search for potential lynchpin 14-3-3-binding phosphosites. The human protein kinome was used as a ‘test data set’ for analysis by ANIA. Protein kinases work with 14-3-3s in two ways: certain protein kinases create 14-3-3-binding phosphosites, and protein kinases that bind to 14-3-3s have also been identified. From ANIA we retrieved the list of 2R-ohnologue protein kinase families. Known and candidate 14-3-3-binding kinases were identified in every subfamily of the human kinome, and potential 14-3-3-binding lynchpin phosphorylated sites were identified. These bioinformatics predictions led to the p21-activated kinases (PAK)4/6/7 protein kinases being experimentally validated as a 14-3-3-binding 2R-ohnologue family.

## Materials and Methods

The list of ‘GD’ mammalian proteins for which 14-3-3-binding sites have been reported (1) was updated by searching PubMed up to October 2013. For a protein to be assigned as a GD, 14-3-3-binding phosphosites had to be shown to be phosphorylated *in vivo* using ^32^P labelling, phospho-specific antibodies and/or mass spectrometry and annotated as phosphorylated in PhosphoSitePlus (18), Phospho.ELM (19) or UniProt (20), and 14-3-3 binding had to be eliminated by dephosphorylation and/or mutagenesis of the candidate sites. Some studies also demonstrated functional correlation between phosphorylation and 14-3-3 binding *in vivo* and *in vitro*, and structural analysis of the target–14-3-3 interaction. A list of ‘silver standards’ was also prepared from the literature, meaning proteins demonstrated to display direct phosphorylation-dependent binding to 14-3-3s, but where phosphorylated residues were not identified. Relevant references are cited in electronic supplementary material (Supplementary Table S1). Each 14-3-3 binding protein was mapped to the human UniProt entry (6 March 2013 release) (20) to be stored in the database.

The list of HT experiments reported in (5) was updated by searching PubMed up to October 2013. The identified proteins were mapped to the human UniProt entry (6 March 2013 release) (20) to be stored in the database. The GD data set and HT data sets can be downloaded from ANIA (https://ania-1433.lifesci.dundee.ac.uk/prediction/webserver/index.py/download). Proteins found to bind non-specifically to Sepharose or agarose beads in control experiments in our laboratory (2) are labeled as ‘contaminants’ in the database, though this does not exclude the possibility that these proteins may also display specific binding to 14-3-3.

The data set of 2R-ohnologue protein families was as described in (1), drawing on data in (21,22). Additionally, the 2R-ohnologue complement of the human protein kinome was manually compiled by performing phylogenetic and gene synteny analyses of 2R-ohnologue families as in (1) (Supplementary Table S2). The *Branchiostoma* and *Ciona* pro-orthologues corresponding to each human 2R-ohnologue protein kinase family were also identified using an automated python script with BLAST over the non-redundant protein sequence database at NCBI (http://blast.ncbi.nlm.nih.gov/Blast.cgi), followed by manual inspection.

To identify candidate lynchpin 14-3-3-binding phosphosites, we first aligned members of each human protein 2R-ohnologue family, together with the corresponding *Branchiostoma* and *Ciona* pro-orthologue of that family. The alignments were performed with five different multiple sequence alignment programs [ClustalO (23), ClustalW2 (24), Mafft (25), Muscle (26) and T-coffee (27)]. This was achieved by compiling a python script to interface with the Representational state transfer web service at the EBI. A python script with Biopython library (28) was used to identify conserved serine and threonine residues in the alignments. Thereafter, candidate 14-3-3-binding serine and threonine residues in the human protein were selected using a prediction algorithm developed in our laboratory (Michele Tinti and the group of Geoff J. Barton, in preparation), by using the GD 14-3-3-binding phosphosites to train a Position-Specific Scoring Matrix and a Neural Network. Predicted sites from both methods were labeled ‘candidate lynchpin’ if the sites are conserved in the human proteins from a given 2R-ohnologue family and also in the *Branchiostoma* or *Ciona* pro-orthologue proteins, and are annotated as phosphorylated in PhosphoSitePlus (18), Phospho.ELM (19) or UniProt (20). We excluded regions annotated in UniProt as ‘extracellular’ and ‘transmembrane’ from these analyses.

Rat and mouse orthologues for the human proteins were also retrieved from InParanoid (29), and by using automated BLAST (80% threshold homology applied).

Additionally, single nucleotide polymorphisms that map to known and predicted 14-3-3-binding phosphosites were retrieved from the catalogue of somatic mutations in cancer (COSMIC) database (www.sanger.ac.uk/genetics/CGP/cosmic/) (30), ontology keywords were retrieved from UniProt (20), and proteins were linked with diseases using the Genetic Association Database (31).

Pre-computed predictions were generated for all human 2R-ohnologue proteins. Results are visualized using embedded Jalview applets (32) to show features of the candidate lynchpins, including sequence conservation, sequence disorder using GlobPlot predictions (33) and where they exist, GD experimentally determined 14-3-3-binding phosphosites are highlighted.

### DAVID analysis

We queried ANIA with the UniProt identifiers (Ids) from the 6 March 2013 release, downloaded the results from the ANIA database and submitted the GD proteins plus 2R-ohnologue proteins containing candidate lynchpins to the DAVID Database for functional annotation clustering with high stringency. We similarly analysed a second list of UniProt Ids from which kinases were filtered out.

### Biochemical analyses

PAK4, PAK6 and PAK7 cDNAs (coding for Genbank sequences O96013, Q9NQU5 and Q9P286, respectively) were amplified from IMAGE consortium EST clones 3 867 403, 5 170 347 and 4 821 164, respectively. The vector for expression of proteins in HEK293 cells was pcDNA5 FRT/TO that adds a C-terminal GFP tag to the expressed protein. PAK4 cDNA was cloned into this plasmid as a BamHI/NotI insert at the multiple cloning site just upstream of, and in frame with, the GFP coding sequence. PAK6 and PAK7 cDNAs were cloned into the same vector as EcoRI/NotI inserts. Polymerase chain reaction and mutagenesis reactions were carried out using KOD Hot Start DNA polymerase (Novagen), and DNA sequencing was performed by The Sequencing Service, College of Life Sciences, University of Dundee.

For biochemical analyses of the PAK protein kinases, HEK293 cells cultured in medium containing 10% (v/v) fetal bovine serum were transfected to express the indicated GFP-tagged proteins. Where indicated, after 24 h, cells were serum-starved for 10 h, then stimulated with, phorbol-12-myristate-13-acetate (PMA, 100 nM for 30 min). PAK-GFP proteins were isolated from cell lysates (2.5 mg) using GFP-Trap® agarose (Chromotek), and washed thrice with lysis buffer [40 mM Tris–HCl (pH 7.5), 1 mM EDTA, 1 mM EGTA, 1% (v/v) Triton X-100, 1 mM sodium orthovanadate, 10 mM sodium β-glycerophosphate, 50 mM sodium fluoride, 5 mM sodium pyrophosphate, 0.27 M sucrose, 1 µM microcystin-LR, 0.1% 2-mercaptoethanol, 1 mM benzamidine, 0.2 mM PMSF and one ‘Complete’ protease inhibitor cocktail tablet (Roche) per 50 ml]. The isolated proteins were tested for their ability to interact directly with 14-3-3 in Far-Western assays, in which digoxygenin-labelled 14-3-3 (a mix of the *Saccharomyces cerevisiae* BMH1 and BMH2 14-3-3 isoforms) take the place of the primary antibody that would be used in a conventional western blot (12). 14-3-3 proteins from the HEK293 cell lysate that co-immunoprecipitated with the PAK-GFP proteins were detected with the pan-14-3-3 antibody K19 (Santa Cruz).

For dephosphorylation reactions, GFP-Trap® beads (5 μl, bulked out with 45 μl of Sepharose) were used to isolate GFP-tagged proteins from 2 mg of lysate of transfected HEK293 cells. The beads were washed into reaction buffer [50 mM Tris–HCl, pH 7.5, 100 mM NaCl, 0.1 mM EGTA, 0.01% Brij-35 and 2 mM dithiothreitol (DTT)] and split for three 50-μl reactions. Proteins were dephosphorylated with 3 μg of lambda phosphatase in the presence of 2 mM MnCl_2_, plus 10 mM EDTA for the inhibited control, for 30 min at 30°C. After further washing, beads were resuspended in sodium dodecyl sulphate sample buffer and analysed for retention of copurified endogenous 14-3-3 proteins (K19 pan-14-3-3 antibody), and for the ability of proteins to bind directly to 14-3-3s in Far-Western overlays.

## Results

**Figure 1.**
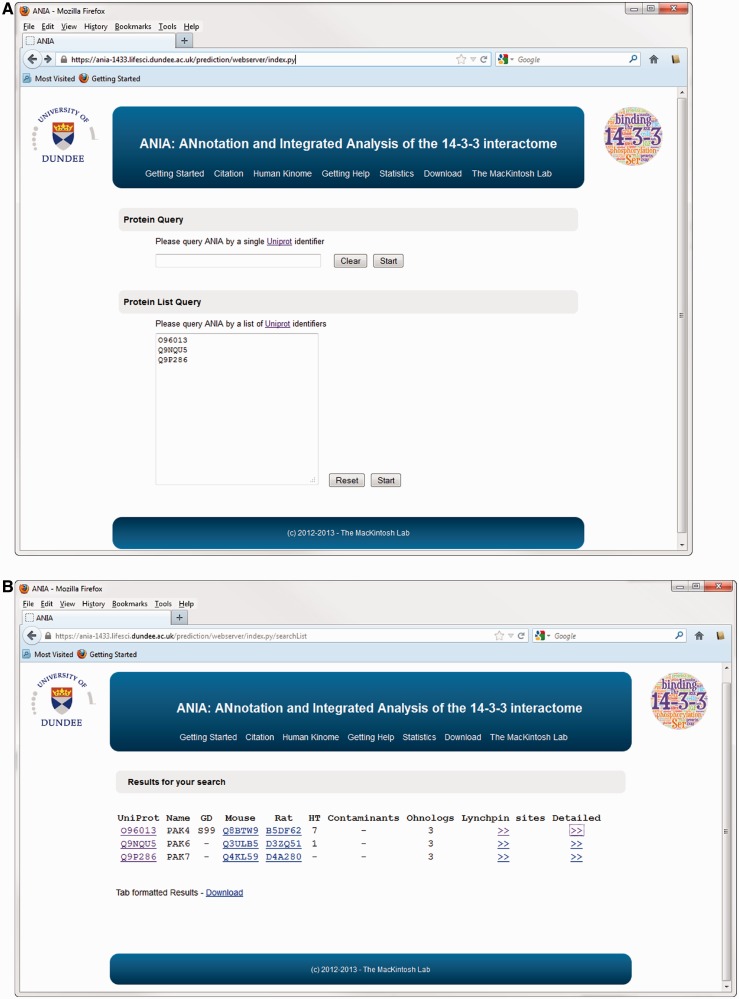
Protein query on ANIA. (**A**) Homepage of ANIA showing the protein and protein list query fields. (**B**) Sample results webpage for the default protein list query. The result page display the UniProt Id (UniProt), the UniProt name (Name) and the presence of a Golden Standard site (GD) on the query proteins. The page also lists the mapping of the input UniProt Ids to the Mouse and Rat UniProt Ids. Under ‘HT’ the page displays the number of HT experiments in which the protein was isolated by 14-3-3-affinity capture, and under ‘contaminants’ is given the number of in-house experiments in which the protein with the input UniProt Ids was isolated by non-specific binding to chromatography matrix with no bound 14-3-3. If the input UniProt Id belongs to a 2R-ohnologue family, the ‘Ohnologs’ column reports the number of protein members of that family. Finally two hyperlinks connect the page to the candidate Lynchpin site prediction (Lynchpin sites) and the detailed information page (Detailed) for the input UniProt Id.

**Figure 2.**
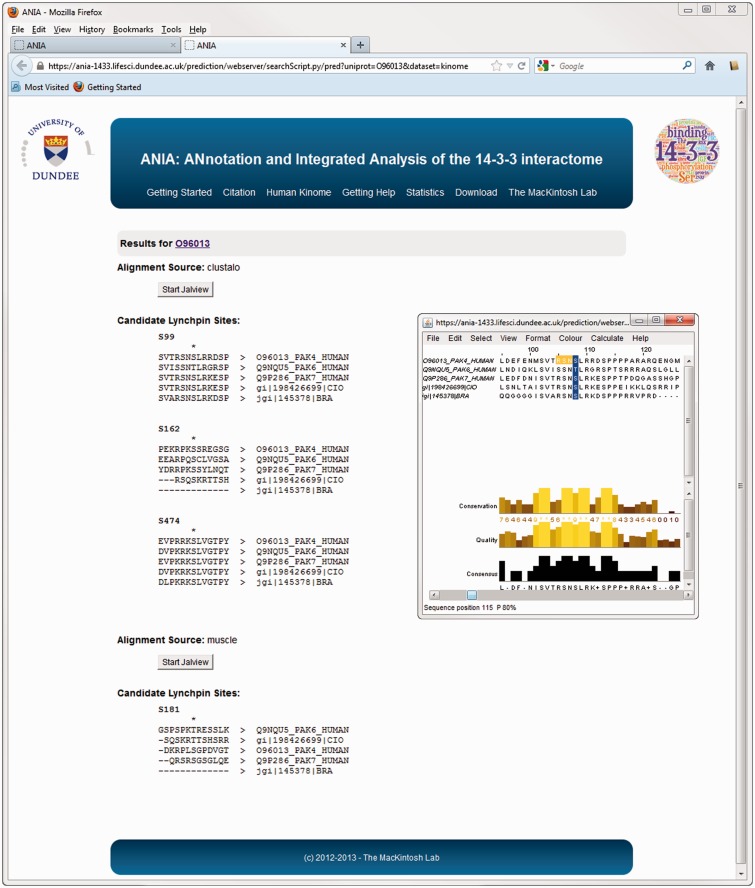
Advanced query results on ANIA. Example of prediction of candidate lynchpin and other 14-3-3-binding phosphosites for the protein kinase PAK4.

**Figure 3.**
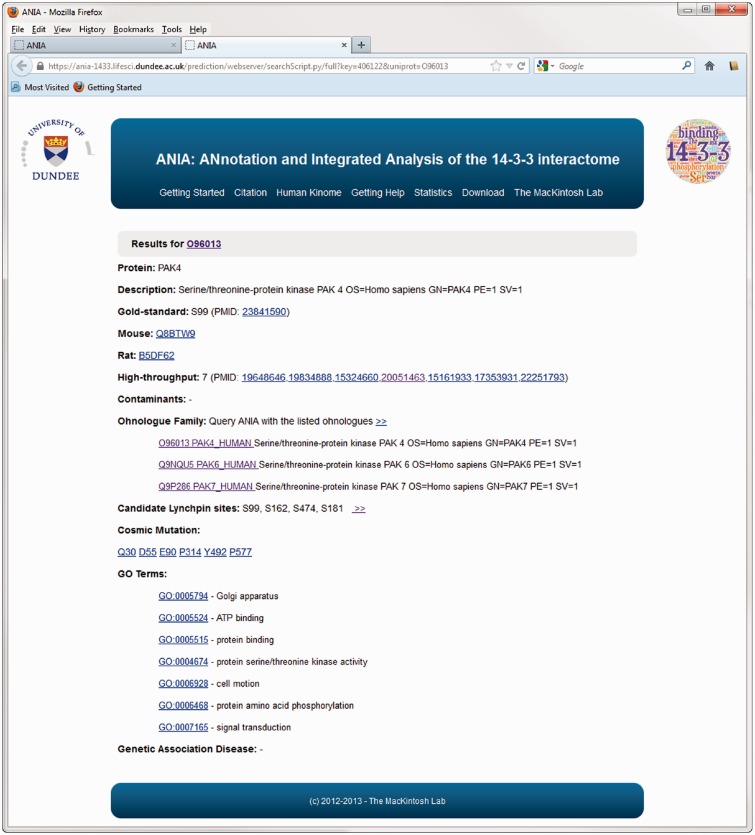
Example of an extended information page for the protein kinase PAK4 in ANIA. These pages include information on somatic cancer mutations, on whether proteins appear in a data set of typical contaminants in HT experiments (from in-house data), and enriched information from UniProt and GO.

The database part of ANIA provides a simple user interface to retrieve information on proteins that have been identified in HT and LT experiments, and 14-3-3-binding phosphosites that have been defined experimentally. Users can search using UniProt identifiers of any human, mouse or rat protein (Figure 1A). Such a search returns a tabular overview of information on the query protein and other members of its 2R-ohnologue family (when relevant), indicating which of these proteins are gold-standard 14-3-3 binders, which exist in data sets of HT 14-3-3 experiments, and a link to pre-computed predictions of candidate 14-3-3-binding sites when available (Figure 1B). From the results overview page, one can link to a ‘Candidate sites’ webpage containing Jalview applets, which enable a richer analysis of the sequence alignments and candidate lynchpin phosphosites (see later example of the PAK kinases). When available, a number in parenthesis to the right of the candidate lynchpin indicates the position of a predicted secondary site that resides after 19 and no more than 40 amino acids from the lynchpin, and which may help to identify the second of a pair of 14-3-3-binding phosphosites on a target (Figure 2): While tandem pairs of phosphosites can bind to the same 14-3-3 dimer if they are a minimum of 15 residues apart, with no maximum, 20–40 residues is a typical gap between two sites within a disordered region (5). Additional meta-data are shown from the ‘Detailed’ link, including information of somatic cancer mutations that map to known and candidate 14-3-3-binding phosphosites, information on whether proteins appear in a data set of typical contaminants in HT experiments [from in-house data, as reported in (1)], and links to data on gene-associated diseases (Figure 3). Additionally, enriched information from UniProt and gene ontology (GO) is provided as well as any cancer polymorphisms annotated in COSMIC. The rationale for providing the cancer polymorphisms information comes from the observation that several 14-3-3 binding regions contain polymorphisms that could in principle interfere with the binding to 14-3-3 (Supplementary Figure S1). In some cases, polymorphisms that disrupt 14-3-3 interactions have been linked in a causative way to disease, and identifying further cases therefore has implications for the discovery of new disease biomarkers and for the development of molecular tools that interfere with 14-3-3-phosphoprotein interactions (34,35). All the queried data can be downloaded from the website as tab-formatted flat files.

Alternatively, a ‘Protein List’ query can be used by submitting a list of human/mouse/rat UniProt identifiers (Figure 1A). Such a search could be used, for example, to compare the results of a new HT experiment with the existing data. In the results page, the protein list queries assemble the result for each submitted protein in a different line (as outlined above for individual protein queries) (Figure 1B).

### Use of ANIA to browse the data from high-thoughput (HT) 14-3-3-affinity capture experiments

**Figure 4.**
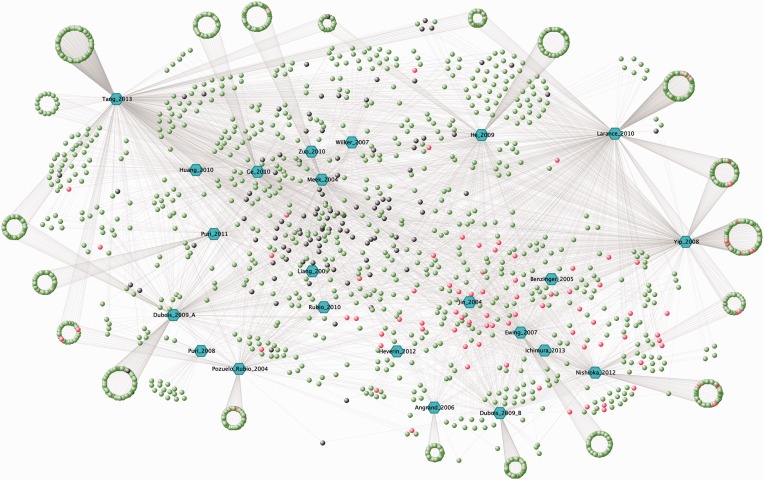
The 14-3-3 interactome from HT 14-3-3-affinity capture experiments. A VisANT graph showing overlaps among the lists of proteins identified for their affinity for 14-3-3s in seven proteomics screens updated to 2013 (Table 1). The input file for the VisANT graph is available in the download section of ANIA. Each paper is assigned a hexagonal node in blue and lines connect the articles to the proteins, which are assigned as green nodes as the default. In red are the GD proteins for which 14-3-3-binding sites were reported in previous LT studies. Proteins from our in-house ‘contaminants’ list (see text) were changed to black.

To date, ANIA contains information from 23 HT experiments in which proteins were captured on immobilized 14-3-3 from extracts of mammalian cells and tissues (https://ania-1433.lifesci.dundee.ac.uk/prediction/webserver/index.py/download). The representation of GDs in these HT data sets is relatively low (3%), which means that much work is needed to distinguish which of the protein hits from the HT experiments are phosphoproteins that bind directly to 14-3-3, and which are indirect binding partners for 14-3-3. To help this effort, we analysed the HT 14-3-3-capture data sets using the filters available in ANIA. First, we selected the seven published HT experiments (Table 1 and blue hexagons in Figure 4) that are most highly enriched in GD 14-3-3-binding proteins (red circles), and the overlaps between the proteins in these HT experiments were visualized in a VisANT graph (36) (Figure 4). We then annotated the proteins from these HT experiments with ANIA, where 14-3-3 binding information on these proteins and other proteins in the same 2R-ohnologue families can be found (Supplementary Tables S2 and S3).

**Table 1.** The list of HT 14-3-3 affinity capture studies in ANIA that have the highest proportion of GD 14-3-3 binding proteins

PMID	First author	Year
16959763	Angrand	2006
15778465	Benzinger	2005
19648646	Dubois	2009
17353931	Ewing	2007
22837806	Heverin	2012
15324660	Jin	2004
22251793	Nishioka	2012

### Tailoring ANIA to analyse the human kinome

**Figure 5.**
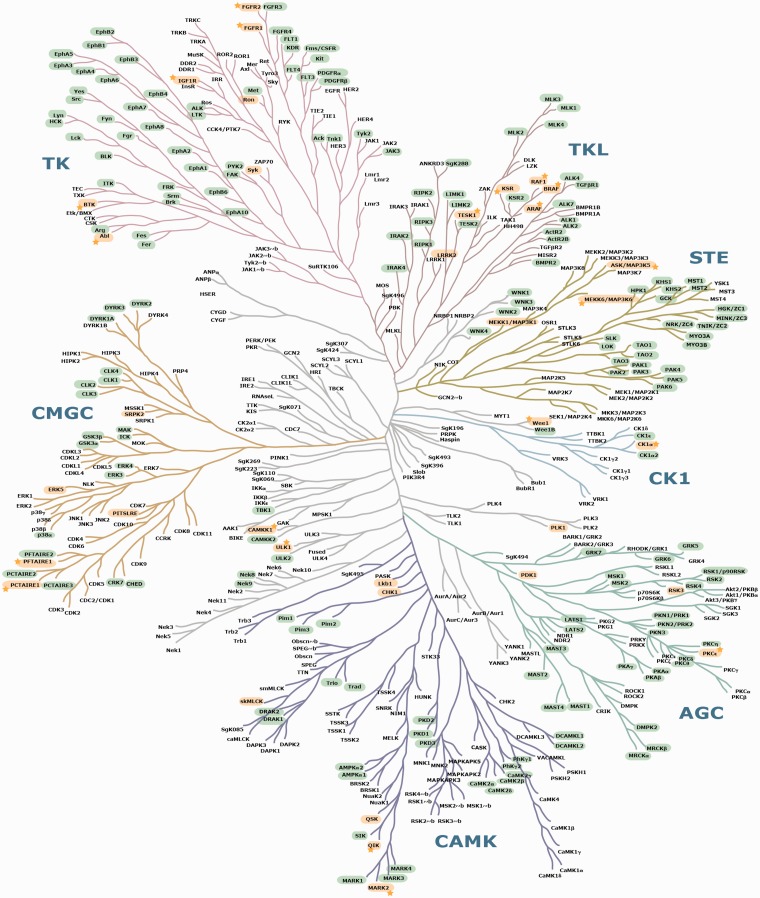
Mapping of known and candidate 14-3-3-binding phosphoproteins on the phylogenetic tree of the human kinome. This image of the human kinome phylogenetic tree is useful because of its familiarity to many researchers (37), though a few further kinases identified since 2002 are also listed in Supplementary Table S2. Protein kinases with phosphosites that conform to candidate 14-3-3-binding sites are highlighted in green. GD 14-3-3 binders are in yellow. Stars are assigned to those GDs for which experimentally determined 14-3-3 phosphosites were also pinpointed by ANIA as being candidate lynchpins. On a hyperlinked version of this figure within ANIA (https://ania-1433.lifesci.dundee.ac.uk/prediction/webserver/index.py/kinome), clicking on a kinase on the dendogram will open the relevant results overview page. Abbreviations: AGC, PKA/PKG/PKC-family kinases; CAMK, calcium/calmodulin-dependent kinases; CK1, casein kinases; CMGC, CDK/MAPK/GSK3/CLK-family kinases; RCG, receptor guanylate cyclases; STE, sterile homologue kinases; TK, tyrosine kinases; TKL, tyrosine kinase-like kinases; atypical protein kinases; Other, belonging to none of the mentioned groups. The human kinome dendogram was adapted from (37).

The eukaryotic protein kinase (ePK) superfamily comprises 477 protein kinases in humans (37) (Figure 5 and Supplementary Table S2) that regulate nearly every process in the cell. Dysregulated kinase activities are cause or consequence in cancer and other diseases, and these enzymes are therefore major targets for therapeutic interventions. However, regulatory interactions and feedback mechanisms among different protein kinases mean that kinase inhibitors often have wider effects than anticipated, and kinase–kinase interactions can also mediate drug resistance mechanisms (38).

Among the 7120 human proteins in 2419 2R-ohnologue protein families that were assembled for ANIA, there are 348 ePK protein kinases in 123 2R-ohnologue families (Figures 4 and 5, and Supplementary Table S2). Furthermore, 28 2R-ohnologues and 9 non-ohnologues of the human kinome are known 14-3-3 binders, and these are distributed in every major branch of the kinome tree (Figure 5). The new paradigm of signal multiplexing by 14-3-3s might therefore help explain many of the regulatory interactions among protein kinases that are confounding therapeutic strategies. To assist further analyses, we therefore performed lynchpin predictions for the human kinome, pinpointing 53 2R-ohnologue protein kinase families with candidate lynchpins that have not been identified experimentally (Supplementary Table S4). The result of this analysis can be browsed in ANIA with a hyperlinked version of Figure 5 available at https://ania-1433.lifesci.dundee.ac.uk/prediction/webserver/index.py/kinome.

### PAK4, 6 and 7 as novel 14-3-3 binding partners

**Figure 6.**
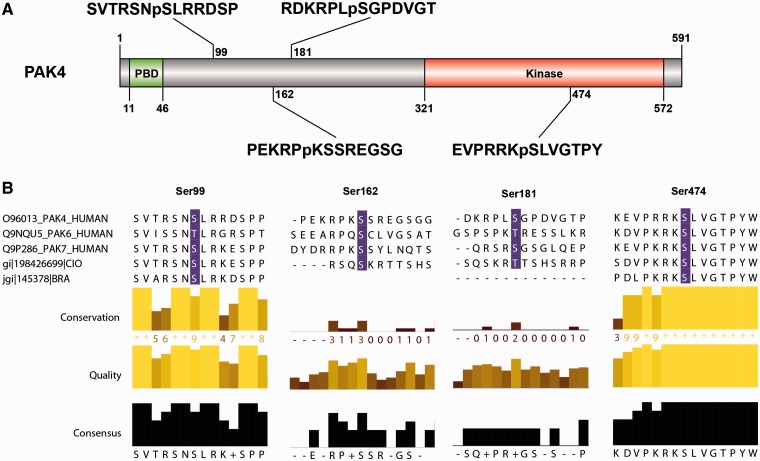
PAK4 sequence features and 14-3-3-binding sites. (**A**) The sequence features of PAK4 (1–591) include a p21-binding domain (PBD) containing a Cdc42/Rac interactive binding domain (CRIB), five ‘low complexity’ regions, a guanine nucleotide exchange factor-interaction domain (GEF-ID) and a protein kinase domain (in red). (B) The sequences around the four candidate 14-3-3-binding sites identified by ANIA (Ser99, Ser162, Ser181 and Ser474) are shown, with the relative conservation of the sequences, comparing PAK4, PAK6 and PAK7 (which belong to the same 2R-ohnologue family) illustrated using Jalview. Note that Ser474 in the kinase activation loop must be phosphorylated for PAK4 to be active, and was therefore provisionally rejected as a likely lynchpin. Protein names conform to UniProt nomenclature: some authors refer to PAK6 as PAK5, while others also refer to PAK7 as PAK5.

As an example, when ANIA was queried about PAK4, 6 and 7 in the type II p21-activated kinase family (UniProt O96013, Q9NQU5 and Q9P286, respectively), both PAK4 and PAK6 have been captured by 14-3-3s in HT experiments (Figure 1B), and PAK4 is a GD, with phosphoSer99 reported as a 14-3-3-binding phosphosite (39). Interestingly, COSMIC reports a Ser99Phe mutation of PAK7 in a case of malignant melanoma, and ANIA predicts Ser99 as a candidate ‘lynchpin’ 14-3-3 binding phosphosite, which is conserved in human PAK4, 6 and 7, as well as the *Branchiostoma* and *Ciona* pro-orthologues. On PAK4, ANIA also pinpoints Ser162, Ser181 and Ser474 for attention as candidate 14-3-3 binding sites (Figures 2 and 6, and Supplementary Tables S2 and S3). Of these, Ser474 is in the activation loop of the kinase domain and its phosphorylation is required for PAK4 activity (40). Its conservation is therefore likely to be linked to its kinase-activating role, and its position within a functional domain does not conform to the norm for 14-3-3-binding sites.

**Figure 7.**
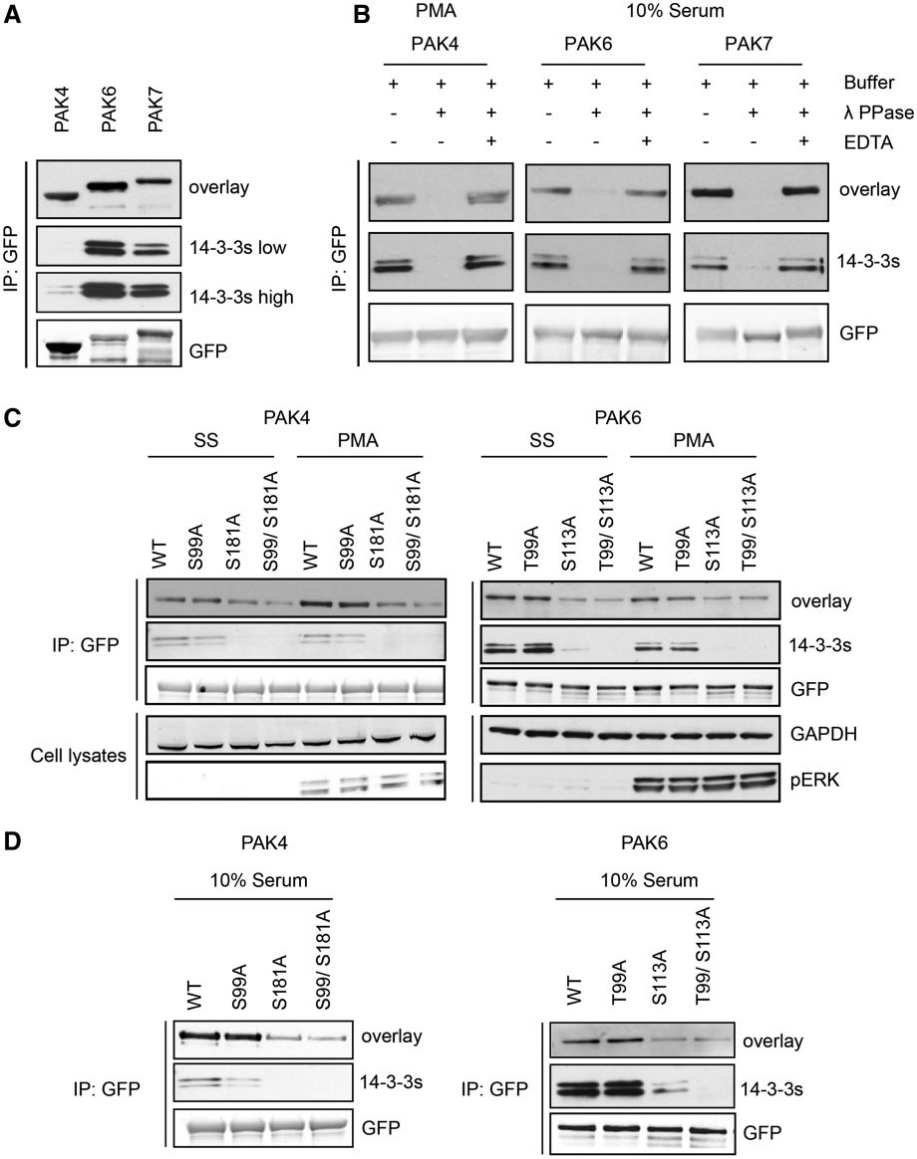
Differential binding of 14-3-3s to overexpressed PAK4-GFP mutants. (**A**) HEK293 cells growing in media containing 10% serum were transfected to express PAK4-GFP, PAK6-GFP and PAK7-GFP. GFP-tagged proteins isolated from cell lysates (∼20 mg) with GFP-Trap® were tested for their ability to bind directly to 14-3-3s in Far-Western assays (overlay) and by co-immunoprecipitation of endogenous 14-3-3s (14-3-3s). Anti-GFP signals show levels of the tagged kinases in the immunoprecipitates. (**B**) HEK293 cells transfected to express the GFP-tagged proteins were serum-stimulated, or serum-starved for 10 h and stimulated with PMA, as indicated. The GFP-tagged proteins bound to GFP-Trap® were dephosphorylated with lambda phosphatase, or not when the phosphatase was inhibited with EDTA. The immunoprecipitates were washed, and analysed for their ability to bind directly to 14-3-3s (overlay), and for retention of co-purified endogenous 14-3-3 proteins (K19 pan-14-3-3 antibody). (**C**) After transfection to express wild-type and mutants PAK4-GFP and PAK6-GFP proteins, HEK293 cells were serum-stimulated, or serum-starved for 10 h and stimulated with PMA, as indicated. The isolated proteins were tested for binding to 14-3-3s in a Far-Western assay. Co-immunoprecipitating endogenous 14-3-3s were detected using the K19 pan-14-3-3 antibody. For the cell lysates, GAPDH was loading control, and the efficacy of PMA stimulation of cells was monitored with the pERK antibody. (**D**) HEK293 cells were transfected to express wild-type and mutant forms of PAK4-GFP and PAK6-GFP. Proteins were immunoprecipitated from lysates with anti-GFP and tested for 14-3-3 binding (overlay) and co-immunoprecipitation of endogenous 14-3-3 (14-3-3). The anti-GFP antibody signals indicate total protein levels.

Experimentally, PAK4, PAK6 and PAK7 isolated from transfected HEK293 cells could bind directly to 14-3-3, with PAK6 and PAK7 displaying the strongest signals (Figure 7A). The interaction of 14-3-3s with PAK4, PAK6 and PAK7 is phosphorylation dependent (Figure 7B) and markedly enhanced for the PAK4 kinase when cells were stimulated with PMA (Figure 7B and C). Ser162Ala and Ser474Ala mutations had no obvious effect on 14-3-3 binding to PAK4 under any conditions tested (not shown). However, 14-3-3 binding was decreased by Ser99Ala and Ser181Ala mutations, with the Ser181Ala having the more pronounced inhibitory effect (Figure 7C and D). These findings indicate that phosphoSer99 (TRSN(pS99)LR) and the less-conserved phosphoSer181 (KRPL(pS181)GP) mediate interaction of PAK4 with a 14-3-3 dimer. For PAK6, phorbol ester gave no enhancement of 14-3-3 binding (Figure 7C). Of the six residues highlighted by ANIA in PAK6, mutagenesis indicated that Thr99 that aligns with Ser99 of PAK4 has a minor effect on 14-3-3 binding, with a greater contribution from phosphoSer113 [RRAQ(pS113)LG] that is not conserved in other members of this protein family (Figure 7C).

### Proteome-wide prediction of 14-3-3 binding proteins

**Figure 8.**
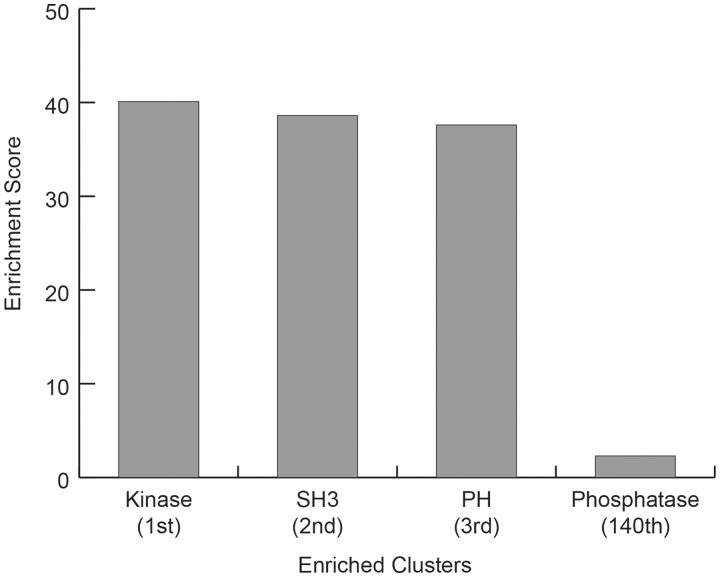
DAVID Enrichment Analysis for GD 14-3-3 binding proteins plus 2R-ohnologues with candidate 14-3-3-binding lynchpin phosphosites. For the GD 14-3-3-binding proteins plus 2R-ohnologues with candidate 14-3-3-binding lynchpins, are given the DAVID (Database for Annotation, Visualization and Integrated Discovery) enrichment scores relative to the entire human proteome, for the Top 3 classes (Kinase, SH3 and PH domain containing proteins) and the score for the first identified DAVID class containing the word phosphatase (Phosphatase).

The ANIA database contains lynchpin predictions for 2963 proteins. To analyse the landscape of the most enriched protein classes with known and predicted lynchpins, we used the DAVID database (41). As illustrated in Figure 8, the top three enriched protein classes identified with the DAVID resources are kinases, and proteins containing SH3 and PH domains. Interestingly, the first cluster of known and predicted 14-3-3-binding proteins containing phosphatase domains appear only at position 140 of the DAVID ranking system (Figure 8 and Supplementary Table S4), which may reflect that phosphatases are often regulated via regulatory subunits, some of which are known 14-3-3-binding phosphoproteins. To exclude a bias towards the protein kinases that were manually curated for the lynchpin site predictions, we repeated the DAVID enrichment analysis after filtering out the protein kinase UniProt identifiers. Also in this case, the top enriched classes contained SH3 and PH protein adaptor modules (Supplementary Table S5).

## Discussion

The >4000 publications on 14-3-3s listed in PubMed reflect the pleiotropic roles of these proteins in engaging with diverse phosphoproteins to regulate metabolism, signal transduction, gene expression, cytoskeletal dynamics, vesicle and protein trafficking, cell cycle, cell survival, growth and proliferation. These include mammalian proteins deregulated in diabetes, cancer and neurological disorders. Here, we provide ANIA as a convenient pipeline for bench scientists and clinicians to find and analyse the >2000 mammalian proteins that have been identified in LT and HT 14-3-3-capture experiments. ANIA stores the human 14-3-3 binding partners and maps each interaction to the mouse and rat proteins, as these are commonly used models for human studies. As well as providing a searchable database for known and candidate 14-3-3-binding proteins and phosphosites, ANIA performs sequence alignments of 2R-ohnologue families and incorporates an algorithm that pinpoints potential 14-3-3-binding phosphosites. These putative sites provide a guide to accelerate experimental validation experiments. The experimenter should also take further criteria into account, such as the minimum distance on a target protein that could reach from one binding pocket to the other within the central groove of a 14-3-3 dimer (∼15 amino acid residues), and we therefore embedded a preliminary search for second sites in ANIA. Special characteristics and structural information about the target under study can also be considered. For example, in the case of the protein kinases, ANIA highlights activation loop phosphorylations within the catalytic centres of the enzymes for attention as potential 14-3-3-binding phosphosites. While perhaps premature to designate activation loop phosphorylations as an ‘excluded group’ in ANIA, 14-3-3 binding to such sites would probably not be compatible with kinase activity. Overall therefore, ANIA offers a new tool whose value in making experiments faster and more precise will be enhanced when experimenters draw on specialized knowledge of their target proteins.

Here, we used ANIA to study the human kinome, and our predictions indicate that 14-3-3s may interact with up to 40% of human kinases. This tentative figure includes the >30 protein kinases known to bind directly to 14-3-3 (including this study), cases where known 14-3-3-binding sites are conserved in other members of the same 2R-ohnologue protein kinase family, and protein kinases that display affinity for 14-3-3 in HT experiments. It would be fascinating to have a complete map of all the protein kinases that bind to 14-3-3s, their 14-3-3-binding phosphosites and the protein kinases that phosphorylate them. Such a connectivity diagram would provide a logical framework to help understand complexities of kinase signaling such as feedback mechanisms and certain unexpected pleiotropic effects of protein kinase inhibitors. 14-3-3-binding phosphosites are generally created by protein kinases in the AGC and CAMK subfamilies. AGC and CAMK enzymes are therefore likely to be direct upstream regulators of the many other protein kinases that are known or inferred to be part of the 14-3-3-interactome. To understand the functioning of the kinase network, we must define these regulatory interconnections and how they co-evolved with the signaling network as a whole.

In common with other classes of signaling proteins, the human protein kinome is enriched in 2R-ohnologue protein families (1,16). Comparing the patterns of 14-3-3-binding sites in different protein kinase 2R-ohnologues families should therefore give insights into how the 2R-WGD expansion of the protein kinome contributed to the evolution of the vertebrate animals. Note that closely related enzymes are not always 2R-ohnologues of each other. For example, *Branchiostoma* pro-orthologues have now been identified for both LRRK1 and for LRKK2 in the tyrosine-kinase-like (TLK) branch of the kinome, which suggests that both enzymes existed before the 2R-WGD.

While ANIA facilitates mapping of the 14-3-3-interacting kinome, we emphasize that only the type of biochemical analyses performed here for the PAK can define 14-3-3-binding phosphosites with confidence. There are six PAKs in two 2R-ohnologue families (corresponding to group I and II PAKs), and interestingly, all six were predicted as candidate 14-3-3-binding targets, though only PAK4 had previously been experimentally determined as such. Here, ANIA short-listed candidate serine and threonine residues, which led to phosphoSer181 being experimentally identified as the major site responsible for the binding of PAK4 to 14-3-3, in addition to the previously identified phosphoSer99 (37). Thus the ‘lynchpin’ (phosphoSer99) need not be the site with highest 14-3-3 binding affinity. These data are consistent with the identification of phosphoSer181 of PAK4 in recent mass spectrometry-based phosphoproteomic studies of proteins from melanoma cells (42), mouse squamous cell carcinoma (43) and activated Natural Killer cells (44). In contrast to PAK4, the stronger binding of PAK6 to 14-3-3 is not stimulated by phorbol ester. The interaction with PAK6 involves Thr99, which aligns with Ser99 of PAK4, though the much greater contribution is from phosphoSer113. Pinpointing these sites will facilitate studies to define how differential 14-3-3 regulation of the PAK kinase family contributes to coordination of cell morphology and other regulatory processes (40,41).

Interestingly, the analysis of proteins with candidate lynchpin sites showed an enrichment of kinase domains, with SH3- and PH-domain proteins ranked next. These findings suggest that 14-3-3 proteins affect phosphorylation-based signaling networks directly at the kinases and indirectly by modulating adaptor proteins, for example, by 14-3-3–mediated masking of the SH3 or PH domains (45,46). Our results suggest that such mechanisms are more widespread than previously identified.

In summary, ANIA provides an accessible interface to enable anyone to (i) search experimental 14-3-3-interactome data sets, and (ii) use algorithms and 2R-ohnologue family sequence alignments to improve the prediction of 14-3-3-binding proteins and phosphosites. Combining data on binding specificity with evolutionary perspective should make ANIA a powerful tool for accelerating the discovery of 14-3-3-based regulatory mechanisms, and learning how the whole 14-3-3-interactome works in health and disease.

### Update process

We welcome participation and feedback from the scientific community to improve ANIA, and aim to update ANIA periodically as further data become available.

## Supplementary Data

Supplementary data are available at *Database* Online.
